# Michael Addition Reaction Catalyzed by Imidazolium Chloride to Protect Amino Groups and Construct Medium Ring Heterocycles

**DOI:** 10.3390/molecules24234224

**Published:** 2019-11-20

**Authors:** Zeshu Dai, Qingqiang Tian, Yanwu Li, Suqin Shang, Wen Luo, Xuetong Wang, Dan Li, Ying Zhang, Zhiyao Li, Jianyong Yuan

**Affiliations:** Department of Medicinal Chemistry, College of Pharmacy, Chongqing Medical University, Chongqing 400016, China; daizs11@163.com (Z.D.); Tqingqiang@163.com (Q.T.); lyanwu@163.com (Y.L.); ssqhcq@163.com (S.S.); Lwen1129@163.com (W.L.); wxy998298@163.com (X.W.); ld28115@163.com (D.L.); yingzhang0728@163.com (Y.Z.); 18883850724@163.com (Z.L.)

**Keywords:** imidazolium chloride, amino protection, Michael addition reaction, amino deprotection, solvent-free, seven-membered ring

## Abstract

An effective approach for amino protection and construction of a seven-membered ring has been developed. The method uses imidazolium chloride to carry out the Michael addition reaction at low temperatures and perform amino deprotection or construction of a seven-membered ring at high temperatures.

## 1. Introduction

Many pharmaceutical intermediates or active molecules often contain amino groups. However, the amino groups are unstable and easily oxidized. The protection of amino groups is therefore often needed in the process of drug synthesis. Methods of amino functional group protection have been reported by related literatures or patents [1]. Traditionally, functional groups were protected via amines reacting with CBZ-Cl [2,3], (Boc)_2_O [4,5,6], Fmoc-Cl [7], TFA [8,9] and Alloc-Cl [10] in the presence of inorganic bases. Furthermore, we have found that bases are mainly used as a promoter. Recently, Jadhav et al. [11] reported the use of microwaves (300 W) for selective tert-butoxycarbonylation of various amines. In 2015, Liguori et al. [12] reported the use of 1-butyl-3-methylimidazolium tetrauoroborate [Bmim][BF_4_] as the ionic liquid medium to promote selective Fmoc and Cbz-protection of various structurally amines. Among the relevant protective groups used for amino function, it would also be necessary to mention the sulfonamido [13,14] protecting groups. Several protocols have been developed for the protection of amino groups with substituted sulfonyl chlorides (such as 4-toluolsulfonyl chloride) using various metal-based catalyst reaction conditions. In 2013, Nardi et al. [15] reported a new and eco-friendly process for ferrier glycosylation of sulfonamides and amino acids with various N-nucleophiles under erbium (III) trifluoromethane-sulfonate as a Lewis acid catalyst. However, these catalysts present a lot of disadvantages. Most of these reactions have limitations such as high cost, corrosiveness, and toxicity. Although methods for the protection of various amino groups are well known and well documented in the literature, it is still very important to study the methods of protecting amino groups. Furthermore, in recent years, a number of researchers have focused on developing greener methods.

When we began to study the reaction of o-phenylenediamine with *N*,*N*-dimethylacrylamide catalyzed by imidazolium chloride to construct a seven-membered ring [16,17,18], we found that the reactants reacted in the form of Michael addition [19,20] reaction at 120 °C, firstly. However, when the temperature was raised to 150 °C, the reaction product did not contain any seven-membered ring. After identification, it was one of the raw materials that was formed by the C-N bond cleavaging. In the next study, we find that reaction of o-phenylenediamine with acrylamide in the presence of imidazole hydrochloride resulted in the formation of 1,3,4,5-tetrahydro-2H-benzo[b][1,4]diazepin-2-one. In 2012, Dabiri, M. et al. [21] reported that [Hmim]TFA catalyzed the hetero-Michael reaction of 2-aminothiophenol and acrylate to construct a seven-membered ring. However, this method only provided a ring-closing reaction of 2-aminothiophenol and acrylate, but the ring-closing reaction of substituted phenylenediamine and a variety of Michael acceptors is still not clear.

Michael addition was one of the most important carbon-carbon and carbon-heteroatom bond-forming strategies in synthetic organic chemistry. In the past few years, a myriad array of metal-centered Lewis acid catalysts have been developed, including cadmium (II) chloride [22], ammonium nitrate(V) [23], zirconium(IV) chloride [24], silica-supported aluminum chloride [25], VO(OTf)_2_ [26] and lanthanum trichloride (LaCl_3_) [27]. Recently, green organic chemistry has begun to attract attention in organic synthesis. For example, metal organic frameworks [28] and basic alumina [29]. Although these reactions work well, most of these catalysts were non-recyclable or transition metal salt catalysts. In addition, in some cases, toxic solvents were required, and a long reaction time was needed. Therefore, it was necessary to explore non-metallic catalysts in organic synthesis. We recently reported an environmentally-friendly procedure to efficiently obtain Aza-Michael adducts using imidazolium chloride as a green catalyst.

**Scheme 1:**
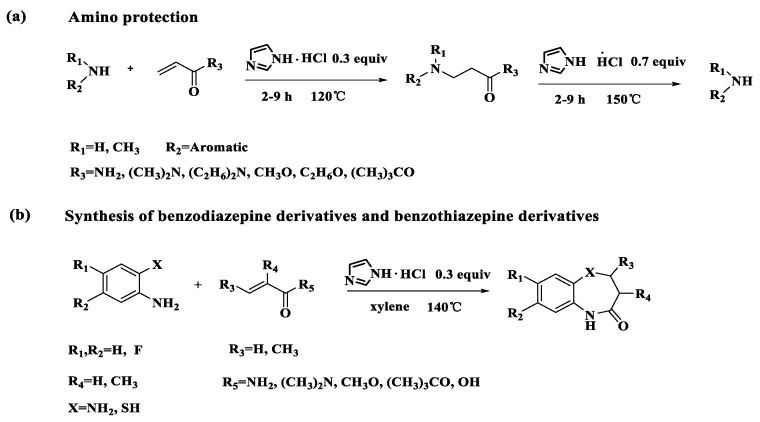
Amino protection catalyzed by imidazole hydrochloride.

Based on these results, we propose using imidazolium hydrochloride to catalyze the Michael addition reaction to protect amino groups and synthesize benzodiazepine derivatives and benzothiazide derivatives. (Scheme 1).

## 2. Results and Discussion

### 2.1. Optimization of Reaction Conditions

In our initial experiments, we choose aniline with *N*,*N*-dimethylacrylamide as a model substrate and the results for development are displayed in Table 1.

Firstly, our investigation commenced with the reaction of aniline **1** and *N*, *N*-dimethyl acrylamide **2** with imidazolium chloride (0.3 equiv) at 110 °C solvent-free. We obtained product **3** (Table 1, Entry 2) in a yield of 69%. However, only a trace product was detected in the absence of catalysts (Table 1, Entry 1). On the other hand, when we used 0.3 equiv of HCl and imidazole as catalysts, we obtained products **3** and **4** (Table 1, Entries 3 and 4) in yields of 60% and 10%, respectively. Inspired by the results, the impact of varying the reaction temperature was investigated (Table 1, Entries 5–8). The best yield was obtained under solvent-free conditions at 120 °C (Table 1, Entry 6). When the temperature was raised or decreased, the yields lowered (Table 1, Entries 5, 7 and 8). These experiments demonstrated that the reaction was highly sensitive to temperature. Next, concerning the amount of catalysts, it was observed that 0.3 eq of imidazolium chloride were sufficient to obtain a good yield. Lowering the amount of catalyst from 30 mol% to 10 mol% resulted in a yield of 32%, indicating that the yields were significantly decreased (Table 1, Entries 9 and 10). Increasing the amount of catalysts had little influence on the yield (Table 1, Entry 11). Finally, we turn our target to the effect of solvents on the reaction. We had tried various solvents such as water and acetonitrile, but unsatisfactory yields were obtained, possibly a result from the effect of temperature on the reaction (Table 1, Entries 12 and 13). However, the reaction was attempted in non-polar media such as toluene and xylene, and a total yield of 63%–66% was obtained (Table 1, Entries 14 and 15).

Interestingly, our team unexpectedly discovered that imidazolium chloride could catalyze the C–N bond-cleavaging reactions at 150 °C. So, after the amino group was protected, we began to screen the conditions for amino deprotection. The *N*, *N*-Dimethyl-3-phenylamino-propionamide (Table 2 and Table 3, **3**) was selected as the model reaction for 6 h. The results are listed in Table 2.

At the beginning of our study, we found that the reaction was performed under the conditions of 0.7 equivalent of imidazolium chloride at 150 °C. The expected product was detected (Table 2, Entry 5). However, only a trace product was detected in the absence of catalysts (Table 2, Entry 1). When 0.7 equiv of HCl was used, the yield of the product 1 (Table 2, Entry 3) was 8%. Furthermore, the impact of varying the reaction temperature was investigated. Compared to the yield of 1 at 150 °C (Table 2, entry 5), the yield of the reaction performed at 120 °C was significantly decreased (Table 2, Entry 4). However, when temperature was increased to 160 °C and 180 °C for 6h, the yields were 63% and 65%, respectively (Table 2, Entries 6 and 7). The expected product was obtained at a yield of 23% only when the amount of catalyst was decreased to 10 mol% (Table 2, Entry 8). The yield of product 1 did not increase significantly when the reaction was carried out at the condition of 1 equiv of catalyst (Table 2, Entry 10) compared to 0.7 equiv of catalyst (Table 2, Entries 5). When the reaction was performed in water or benzene, no reaction occurred in the presence of catalyst (Table 2, Entries 11 and 12). The yield of the expected product was 57% when the reaction was conducted in xylene (Table 2, Entry 13). The optimum yield was obtained when the reaction was carried out under solvent free conditions (Table 2, Entry 5).

However, the reaction of o-phenylenediamine with acrylamide at 140 °C for 5 h to obtain 1,3,4,5-tetrahydro-2H-ben-zo[b][1,4]diazepin-2-one gave a very good yield. Inspired by this result, we herein tried to synthesize benzodiazepines and benzothiazides derivatives through the further study of Michael addition reaction. Hence, the o-phenylenediamine (**4**) and acrylamide (**5**) were selected as the substrates for the optimization studies. Gratifyingly, the substrates **4** and **5** could be transformed into product 6 in a yield of 45% by heating in the presence of imidazole hydrochloride in xylene at 140 °C for 5 h (Table 3, Entry 1). Next, a variety of temperatures were explored; it was observed that the desired yields can be obtained only at 140 °C (Table 3, Entries 5–9). Subsequently, several other solvents such as CH_3_CN, toluene, and 2-ethoxyethanol were screened, among which none gave the products in good yields (Table 3, Entries 10–16). Furthermore, yields of 19% and 49% were obtained when 0.1 eq of imidazolium chloride and 0.5eq of imidazolium chloride were used as the catalysts, respectively (Table 3, Entries 17 and 18).

### 2.2. Scope of Substituted Substrate

With the optimized reaction conditions on hand, the scope of the Michael addition reactions was explored using a wide range of amines and acrylamide derivatives, as shown in Table 4. Firstly, aniline reacted with *N*,*N*-dimethylacrylamide to form the corresponding Michael addition product in a yield of 74% (Table 4, **3a**). Aromatic amine substituted with electron-donating groups (e.g., amino, methoxy, or phenyl) at different positions reacted very well with *N*, *N*-dimethylacrylamide, generating the corresponding products in moderate to good yields (Table 4, **3b**–**3e**). Similarly, aromatic amine possessing a weak electron-withdrawing group, such as 4-bromoaniline, underwent a smooth reaction to obtain a medium yield of the corresponding product **3** (Table 4, **3f** and **3g**). However, it is worth noting that aromatic amines substituted by a strong electron withdrawing group did not react with *N*,*N*-dimethylacrylamide (Table 4, **3h**). Moreover, to further investigate the scope of this reaction system, the reactions of different substituted amines and acrylamide derivatives (e.g., acrylamide *N*,*N*-dimethylacrylamide, methyl acrylate, ethyl acrylate or tert-butyl acrylate) were also tested. Aromatic amine bearing either electron-rich or weak electron-deficient substituents all underwent the reactions smoothly to give the expected products in yields of 33% to 88% (Table 4, **3i**–**3v**). However, the representative of the heterocyclic aromatic amines such as 2-aminopyridine could be transformed into the corresponding product in a yield of 33%. The lower yield was ascribed to lower nucleophilicity of heterocyclic aromatic amines (Table 4, **3s**). The reaction of secondary amines with acrylamide was further tested. The secondary arylamine N-methylaniline was converted into the corresponding product in excellent yields (Table 4, **3w**). Encouraged by these satisfying results, aza-Michael addition of various aliphatic amines and acrylamide were performed. The aza-Michael addition of pyrrolidine and 4-methylpiperidine could also proceed smoothly within shorter reaction period, obtaining the corresponding products in good yields (Table 4, **3x** and **3y**). The higher yields might be attributed to the stronger nucleophilicity of aliphatic amines.

After the amino group was protected, substrate ranges of the deprotection of aromatic amino groups were investigated and the results were displayed in Table 5. Heating a solution of *N*,*N*-Dimethyl-3-phenylamino-propionamide (**3aa**) using 0.7 eq of imidazolium chloride at 150 °C without solvent resulted in the formation of aniline in a yield of 63% (Table 5, **1a**). The use of substrates with electron-donating substituents generally resulted in higher yields than the substrates with a weak electron-withdrawing substituent such as Cl, Br (Table 5, **1b**–**1f**). Next, when the dimethylamino substituent was replaced by -NH_2_ or diethylamino, the yields were 35-81% (Table 5, **1g**–**1j**). Meanwhile, R_3_ substituents such as ethoxy and Tert-butoxy groups were tolerable under these optimized conditions. Compound **3bk**–**3bo** gave the corresponding product in medium yields (Table 5, **1k**–**1o**).

Then, we investigated the functional group tolerance on a seven-membered ring. The data were presented in Table 6. O-phenylenediamine and acrylamide derivatives reacted well, leading to the corresponding products in moderate to good yields (Table 6, **6a**–**6d**). It is worth noting that o-phenylenediamine and *N*,*N*-dimethylacrylamide or tert-Butyl acrylate reactions were not observed under these reaction conditions. The disubstituted o-phenylenediamine (such as 4,5-difluoro-2-phenylenediamine) and acrylamide derivatives were compatible in this reaction, obtaining the corresponding products in good yields (Table 6, **6e**). We explored the reaction of 2-aminothiophenol with acrylamide or methacrylamide under standard reaction conditions, providing the corresponding product in yields of 34% and 31% (Table 6, **6f** and **6g**). These low yields might be attributed to more by-products of the Michael addition reaction.

## 3. Conclusions

In conclusion, we have developed a highly efficient and environmentally friendly method for amino protection and seven-membered ring construction. The reactions initiated by aza-Michael reactions to protect the amino group, followed by deprotection of the amino group or transaminated. This method has a wide scope of substrates, providing a wide range of aromatic amines and aliphatic amines. Also, the significance of our finding can reduce the number or amount of organic solvents, potentially toxicity, and hazardous catalysts. It is most important that we have used non-metallic catalysts and compared them with the other metal catalysts. Moreover, the results provide further evidence that it is possible to use this method in the synthesis of bioactive compounds. It is noteworthy that this method is complementary to the previously reported amino protective reactions.

## 4. Materials and Methods

### 4.1. Chemicals and Materials

All reagents were purchased from Meyer Reagent Co., Ltd. (Shanghai, China), Macklin Reagent Co., Ltd. (Shanghai, China), Chongqing Chuandong Chemical Co., Ltd. (Chongqing, China), etc., and used without further purification. ^1^H- and ^13^C-NMR spectra were recorded on a Bruker Avancell NMR (600 MHz) instruments. Chemical shifts were reported in ppm and coupling constants (*J*) in Hz. TLC plates were visualized by exposing UV light or by iodine. Purification of crude compounds and separation of reaction mixtures were carried out by column chromatography using silica gel (200–300 meshes, Shanghai, China). All substrates are known compounds according to the literature.

### 4.2. General Procedures for Michael Addition Reaction

The **1** (5.0 mmol) and **2** (6.5 mmol) in a molar ratio of 1:1.3 reacted with stirring in the presence of imidazolium hydrochloride (1.5 mmol, 0.3 eq). Heating was performed using an oil bath and the reaction was followed up by TLC until completion. After completion of the reaction, the system was cooled. The organic layer was extracted using water (15 mL) and ethyl acetate (20 mL) and dried using anhydrous Na_2_SO_4_, filtered, and concentrated under reduced pressure. The residue was subjected to column chromatography eluting with petroleum ether: ethyl acetate (7:1) mixtures.

#### 4.2.1. Synthesis of 3-(Pyrrolidin-1-yl)propanamide (**3x**)

To a mixture of pyrrolidine (7.0 mmol, 1 equiv) and acrylamide (9.2 mmol, 1.3 equiv), imidazolium hydrochloride (2.1 mmol, 0.3 eq) was added. The mixture was stirred at RT and monitored by TLC. After the reaction completed, the reaction mixture was cooled to allow the products crystallize (ethyl acetate/methanol (5:1)). The crystallized products were filtered and further washed by ethyl acetate, dried, and evaluated by spectral analysis (**3x**).

#### 4.2.2. Synthesis of 3-(4-Methylpiperidin-1-yl)propanamide (**3y**)

To a mixture of 4-methylpiperidine (5.1 mmol, 1 equiv) and acrylamide (9.2 mmol, 1.3 equiv), imidazolium hydrochloride (2.1 mmol, 0.3 eq) was added. The mixture was stirred at 70 °C and monitored by TLC. After the reaction completed, the reaction mixture was cooled to allow the products to crystallize (ethyl acetate/methanol (5:1)). The crystallized products were filtered, and further washed by ethyl acetate, dried, and evaluated by spectral analysis (**3y**).

### 4.3. General Procedure for Amino Deprotection

Placed **3b** (1 mmol, 1 equiv) and imidazolium chloride (0.7 mmol, 0.7 equiv) in a tube-type schlenk flask, stirred at 150 °C, and monitored the reaction progress by TLC. After the reaction completed, the reaction mixture was cooled. The organic layer was extracted using water (15 mL) and ethyl acetate (20 mL) and dried using anhydrous Na_2_SO_4_, then filtered and concentrated under reduced pressure. The residue was subjected to column chromatography eluting with petroleum ether: ethyl acetate (10:1) mixtures.

### 4.4. General Methods for Synthesis of Substituted 1,3,4,5-Tetrahydro-2H-1,5-benzodiazepine-2-ones and Substituted 2,3-Dihydrobenzo[b][1,4]thiazepin-4(5H)-ones

To 20 mL of schlenk tube with a magnetic stir bar we successively added **4a** (3.0 mmol, 1 eq), **5a** (5.1 mmol, 1.7 eq), and imidazolium hydrochloride (0.9 mmol, 0.3 eq). Then we added xylene (1 mL) to the reaction mixture. The mixture was stirred at 140 °C. The progress of the reaction was monitored by TLC. After the reaction completed, water (15 mL) was added to the reaction mixture. The solution was extracted using ethyl acetate (3 × 15 mL) and dried using anhydrous Na_2_SO_4_. The crude product was purified by flash chromatography eluting with petroleum ether: ethyl acetate (5:1) mixtures.

#### Synthesis of 1,3,4,5-Tetrahydro-2H-benzo[b][1,4]diazepin-2-one (**6c**)

To 20 mL of schlenk tube with a magnetic stir bar, o-phenylenediamine (4.6 mmol, 0.5 g, 1 eq), methyl acrylate (7.9 mmol, 0.68 g, 1.7 eq) and imidazolium hydrochloride (1.4 mmol, 0.15 g, 0.3 eq) were successively added. Then xylene (1 mL) was added to the reaction mixture. The mixture was stirred at 90 °C for 5 h and then at 140 °C for 8 h. The progress of the reaction was monitored by TLC. After the reaction completed, water (15 mL) was added to the reaction mixture. The solution was extracted with ethyl acetate (3 × 15 mL) and dried using anhydrous Na_2_SO_4_. The crude product was purified by flash chromatography using silica gel to obtain the corresponding product (**6c**) (Figures S1–S74).

*N,N-Dimethyl-3-phenylamino-propionamide* (**3a**): The product was obtained as a white solid in a yield of 74% (0.34 g), MP: 103–106 °C [30]. ^1^HNMR (600 MHz, CDCl_3_) δ 7.17 (dd, *J* = 8.4, 7.4 Hz, 2H), 6.69 (t, *J* = 7.3 Hz, 1H), 6.64 (d, *J* = 7.7 Hz, 2H), 4.33 (s, 1H), 3.48 (t, *J* = 6.0 Hz, 2H), 2.95 (d, *J* = 3.5 Hz, 6H), 2.59 (t, *J* = 6.0 Hz, 2H).^13^C-NMR (151 MHz, CDCl_3_) δ 171.62, 147.72, 129.29, 117.57, 113.26, 39.58, 37.09, 35.28, 32.20.

*3-(2-Amino-phenylamino)-N,N-dimethyl-propionamide* (**3b**): The product was obtained as a black liquid in a yield of 72% (0.39 g). ^1^H NMR (600 MHz, CDCl_3_) δ 6.84 – 6.75 (m, 1H), 6.75 – 6.64 (m, 3H), 3.59 (s, 2H), 3.46 (t, J = 6.2 Hz, 2H), 2.96 (d, *J* = 9.6 Hz, 6H), 2.63 (t, *J* = 6.1 Hz, 2H). ^13^C-NMR (151 MHz, CDCl_3_) δ171.73, 136.94, 135.20, 120.21, 119.03, 116.33, 112.65, 40.06, 37.11, 35.31, 32.44.

*2-Amino-N-(2-dimethylcarbamoyl-ethyl)-benzamide* (**3c**): The product was obtained as a white solid in a yield of 43% (0.23 g). MP: 134–136 °C. ^1^H NMR (600 MHz, CDCl_3_) δ 8.04 (s, 1H), 7.41 (d, *J* = 7.9 Hz, 1H), 7.34 (t, *J* = 7.8 Hz, 1H), 6.80 (d, *J* = 8.4 Hz, 1H), 6.61 (t, *J* = 7.5 Hz, 1H), 5.84 (s, 2H), 3.55 (t, *J* = 7.3 Hz, 2H), 3.00 (s, 3H), 2.96 (s, 3H), 2.68 (t, *J* = 7.3 Hz, 2H). ^13^C NMR (151 MHz, CDCl_3_) δ 172.03, 171.13, 149.76, 133.62, 128.45, 114.89, 113.43, 112.02, 39.16, 37.25, 35.37, 32.86.

*N,N-Dimethyl-3-(naphthalen-1-ylamino)-propionamide* (**3d**): The product was obtained as a colorless liquid in a yield of 73% (0.52 g). ^1^H-NMR (600 MHz, CDCl_3_) δ 7.77 (d, *J* = 7.9 Hz, 1H), 7.69 (d, *J* = 7.8 Hz, 1H), 7.39 – 7.28 (m, 2H), 7.26 (t, *J* = 7.8 Hz, 1H), 7.18 – 7.10 (m, 1H), 6.57 (d, *J* = 7.5 Hz, 1H), 5.64 – 4.87 (m, 1H), 3.56 (t, *J* = 5.8 Hz, 2H), 2.86 (d, *J* = 6.3 Hz, 6H), 2.62 (t, *J* = 5.8 Hz, 2H).^13^C-NMR (151 MHz, CDCl_3_) δ 171.86, 143.27, 134.42, 128.48, 126.53, 125.77, 124.76, 123.87, 120.40, 117.37, 104.31, 39.73, 37.15, 35.36, 31.92.

*3-(4-Methoxy-phenylamino)-N,N-dimethyl-propionamide* (**3e**): The product was obtained as a white solid in a yield of 81% (0.5 g). MP: 85-87 °C. ^1^H-NMR (600 MHz, CDCl_3_) δ6.78 (d, *J* = 8.6 Hz, 2H), 6.64 (d, *J* = 8.6 Hz, 2H), 3.75 (s, 3H), 3.43 (t, *J* = 5.9 Hz, 2H), 2.96 (d, *J* = 6.5 Hz, 6H), 2.59 (t, *J* = 5.9 Hz, 2H). ^13^C NMR (151 MHz, CDCl_3_) δ 171.72, 152.41, 141.82, 114.92, 114.91, 55.80, 40.83, 37.12, 35.30, 32.18.

*3-(2-Chloro-phenylamino)-N,N-dimethyl-propionamide* (**3f**): The product was obtained as a yellow liquid in a yield of 82% (0.52 g). ^1^H NMR (600 MHz, CDCl_3_) δ 7.24 (dd, *J* = 7.9, 1.5 Hz, 1H), 7.15 – 7.11 (m, 1H), 6.71 (dd, *J* = 8.2, 1.1 Hz, 1H), 6.61 (tt, *J* = 14.6, 7.3 Hz, 1H), 4.92 (s, 1H), 3.54 (t, *J* = 6.4 Hz, 2H), 2.96 (d, *J* = 2.3 Hz, 6H), 2.62 (t, *J* = 6.4 Hz, 2H). ^13^C NMR (151 MHz, CDCl_3_) δ 170.16, 142.73, 128.25, 126.74, 118.56, 116.21, 110.12, 38.30, 36.08, 34.30, 31.33.

*3-(4-Bromo-phenylamino)-N,N-dimethyl-propionamide* (**3g**): The product was obtained as a white solid in a yield of 55% (0.47 g). MP: 110–113 °C. ^1^H NMR (600 MHz, CDCl_3_) δ7.24 (d, *J* = 8.8 Hz, 2H), 6.52 (d, *J* = 8.8 Hz, 2H), 4.65 (s, 1H), 3.44 (t, *J* = 5.9 Hz, 2H), 2.96 (d, *J* = 8.4 Hz, 6H), 2.58 (t, *J* = 5.9 Hz, 2H).^13^C NMR (151 MHz, CDCl_3_) δ 171.46, 146.80, 131.97, 114.83, 109.08, 39.58, 37.11, 35.32, 31.95.

*3-Phenylamino-propionamide* (**3i**): The product was obtained as a white solid in a yield of 84% (0.39 g). MP: 53–56 °C [31]. ^1^H NMR (600 MHz, CDCl_3_) δ7.18 (t, *J* = 7.9 Hz, 2H), 6.73 (t, *J* = 7.3 Hz, 1H), 6.63 (d, *J* = 7.9 Hz, 2H), 5.91 (s, 2H), 3.44 (t, *J* = 6.1 Hz, 2H), 2.49 (t, *J* = 6.1 Hz, 2H). ^13^C NMR (151 MHz, CDCl_3_) δ 174.26, 147.54, 129.38, 118.09, 113.40, 39.99, 34.91.

*3-(4-Bromo-phenylamino)-propionamide* (**3j**): The product was obtained as a white solid in a yield of 79% (0.68 g). MP: 122–125 °C. ^1^H NMR (600 MHz, CDCl_3_) δ7.29 – 7.28 (m, 1H), 7.27 (d, *J* = 2.1 Hz, 1H), 6.64 – 6.47 (m, 2H), 5.57 (d, 2H), 3.48 – 3.41 (m, 2H), 2.59 – 2.50 (m, 2H). ^13^C NMR (151 MHz, CDCl_3_) δ 173.48, 146.05, 132.10, 115.26, 110.16, 40.19, 34.28.

*N,N-Diethyl-3-phenylamino-propionamide* (**3k**)[32]: The product was obtained as a yellow liquid in a yield of 83% (0.39 g). ^1^H NMR (600 MHz, CDCl_3_) δ 7.21 – 7.11 (m, 2H), 6.68 (t, *J* = 7.3 Hz, 1H), 6.64 – 6.61 (m, 2H), 4.10 (m, 1H), 3.46 (t, *J* = 6.2 Hz, 2H), 3.36 (q, *J* = 7.1 Hz, 2H), 3.23 (q, *J* = 7.2 Hz, 2H), 2.57 (t, *J* = 6.2 Hz, 2H), 1.10 (td, *J* = 7.1, 5.6 Hz, 6H). ^13^C NMR (151 MHz, CDCl_3_) δ170.75, 148.04, 129.25, 117.35, 113.18, 41.90, 40.13, 39.75, 32.00, 14.16, 13.11.

*N,N-Diethyl-3-p-tolylamino-propionamide* (**3l**): The product was obtained as a white solid in a yield of 72% (0.39 g). MP: 52–55 °C. ^1^H NMR (600 MHz, CDCl_3_) δ6.98 (d, *J* = 8.3 Hz, 2H), 6.57 (d, *J* = 8.3 Hz, 2H), 3.45 (t, *J* = 6.1 Hz, 2H), 3.37 (q, *J* = 7.1 Hz, 2H), 3.25 (q, *J* = 7.2 Hz, 2H), 2.58 (t, *J* = 6.1 Hz, 2H), 2.23 (s, 3H), 1.11 (td, *J* = 7.1, 3.1 Hz, 6H). ^13^C NMR (151 MHz, CDCl_3_) δ170.77, 145.45, 129.78, 126.96, 113.66, 41.87, 40.41, 40.09, 31.96, 20.41, 14.17, 13.11.

*3-(2-Amino-phenylamino)-N,N-diethyl-propionamide* (**3m**): The product was obtained as a black liquid in a yield of 63% (0.34 g). ^1^H NMR (600 MHz, CDCl_3_) δ 6.82 – 6.77 (m, 1H), 6.75 – 6.69 (m, 3H), 3.85 (s, 2H), 3.47 (t, *J* = 6.2 Hz, 2H), 3.39 (q, *J* = 7.1 Hz, 2H), 3.29 (q, *J* = 7.2 Hz, 2H), 2.65 (t, *J* = 6.2 Hz, 2H), 1.14 (dt, *J* = 13.9, 7.1 Hz, 6H). ^13^C NMR (151 MHz, CDCl_3_) δ170.87, 136.52, 135.44, 120.22, 119.48, 116.43, 113.28, 41.94, 40.62, 40.17, 32.06, 14.21, 13.12.

*3-(3-Chloro-phenylamino)-N,N-diethyl-propionamide* (**3o**): The product was obtained as a white solid in a yield of 76% (0.48 g). MP: 58–61 °C. ^1^H NMR (600 MHz, CDCl_3_) δ 7.04 (t, *J* = 8.0 Hz, 1H), 6.65 – 6.61 (m, 1H), 6.59 (t, *J* = 2.1 Hz, 1H), 6.49 (dd, *J* = 8.2, 2.2 Hz, 1H), 4.58 (m, 1H), 3.49 – 3.41 (m, 2H), 3.37 (q, *J* = 7.1 Hz, 2H), 3.25 (q, *J* = 7.2 Hz, 2H), 2.57 (t, *J* = 6.0 Hz, 2H), 1.18 – 0.99 (m, 6H). ^13^C NMR (151 MHz, CDCl_3_) δ 171.73, 136.94, 135.20, 120.21, 119.03, 116.33, 112.65, 40.06, 37.11, 35.31, 32.44.

*3-Benzylamino-N,N-diethyl-propionamide* (**3p**): The product was obtained as a white solid in a yield of 84% (0.45 g). MP: 119–121 °C. ^1^H NMR (600 MHz, DMSO-d6) δ 9.44 (s, 1H), 7.68 – 7.51 (m, 2H), 7.51 – 7.37 (m, 3H), 4.26 – 4.04 (m, 2H), 3.27 (q, *J* = 7.1 Hz, 4H), 3.05 (t, *J* = 7.1 Hz, 2H), 2.83 (t, *J* = 7.2 Hz, 2H), 1.11 (t, *J* = 7.1 Hz, 3H), 1.02 (t, *J* = 7.1 Hz, 3H). ^13^C NMR (151 MHz, DMSO-d6) δ 168.78, 132.51, 130.46, 129.32, 129.12, 50.27, 43.13, 41.74, 40.38, 40.24, 40.11, 29.02, 14.40, 13.47.

*3-Phenylamino-propionic acid methyl ester* (**3q**): The product was obtained as a white solid in a yield of 88% (0.41 g). MP: 32–35 °C [33]. ^1^H NMR (600 MHz, CDCl_3_) δ 7.17 (t, *J* = 7.6 Hz, 2H), 6.71 (t, *J* = 7.3 Hz, 1H), 6.61 (d, *J* = 8.1 Hz, 2H), 4.02 (s, 1H), 3.68 (s, 3H), 3.43 (t, *J* = 6.4 Hz, 2H), 2.60 (t, *J* = 6.4 Hz, 2H). ^13^C NMR (151 MHz, CDCl_3_) δ 172.85, 147.60, 129.52, 129.35, 129.15, 117.91, 117.75, 117.54, 113.24, 113.06, 112.83, 99.99, 51.78, 39.43, 33.73.

*3-p-Tolylamino-propionic acid ethyl ester* (**3r**) [34]: The product was obtained as a yellow liquid in a yield of 82% (0.44 g). ^1^H NMR (600 MHz, CDCl_3_) δ 7.02 – 6.91 (m, 2H), 6.58 – 6.51 (m, 2H), 4.14 (qd, *J* = 7.5, 2.3 Hz, 2H), 3.85 (s, 1H), 3.41 (td, *J* = 6.4, 1.0 Hz, 2H), 2.58 (td, *J* = 6.4, 0.8 Hz, 2H), 2.23 (d, *J* = 2.3 Hz, 3H), 1.25 (ddd, *J* = 7.1, 4.5, 1.8 Hz, 3H). ^13^C NMR (151 MHz, CDCl_3_) δ 172.44, 145.32, 129.96, 129.78, 126.92, 113.30, 60.56, 60.52, 39.83, 33.92, 20.37, 20.19, 14.19.

*3-(Pyridin-2-ylamino)-propionic acid ethyl ester* (**3s**): The product was obtained as a grey solid in a yield of 33% (0.15 g). MP: 46–48 °C [35]. ^1^H NMR (600 MHz, CDCl_3_) δ 8.08 (dd, *J* = 4.8, 0.9 Hz, 1H), 7.45 – 7.31 (m, 1H), 6.56 (dd, *J* = 6.7, 5.4 Hz, 1H), 6.40 (d, *J* = 8.4 Hz, 1H), 4.96 (s, 1H), 4.15 (q, *J* = 7.1 Hz, 2H), 3.64 (d, *J* = 5.1 Hz, 2H), 2.63 (t, *J* = 6.2 Hz, 2H), 1.26 (t, *J* = 7.1 Hz, 3H). ^13^C NMR (151 MHz, CDCl_3_) δ 172.61, 158.22, 147.93, 137.37, 112.94, 107.64, 60.60, 37.36, 34.23, 14.20.

*3-p-Tolylamino-propionic acid tert-butyl ester* (**3t**): The product was obtained as a yellow liquid in a yield of 82% (0.44 g). ^1^H NMR (600 MHz, CDCl_3_) δ6.91 (d, *J* = 7.9 Hz, 2H), 6.48 (d, *J* = 8.2 Hz, 2H), 3.83 (s, 1H), 3.29 (t, *J* = 6.3 Hz, 2H), 2.43 (t, *J* = 6.3 Hz, 2H), 2.16 (s, 3H), 1.37 (s, 9H). ^13^C NMR (151 MHz, CDCl_3_) δ 170.79, 144.49, 128.74, 125.82, 112.29, 79.75, 39.02, 34.08, 28.67, 27.09, 26.95, 19.35.

*3-Phenylamino-propionic acid tert-butyl ester* (**3u**) [36]: The product was obtained as a yellow liquid in a yield of 80% (0.37 g). ^1^H NMR (600 MHz, CDCl_3_) δ 7.16 (dd, *J* = 11.1, 4.2 Hz, 2H), 6.69 (t, *J* = 7.3 Hz, 1H), 6.60 (d, *J* = 7.9 Hz, 2H), 4.03 (s, 1H), 3.37 (t, *J* = 6.3 Hz, 2H), 2.50 (t, *J* = 6.3 Hz, 2H), 1.44 (s, 9H). ^13^C NMR (151 MHz, CDCl_3_) δ 171.79, 147.88, 129.33, 129.30, 117.62, 117.59, 113.10, 113.07, 80.86, 39.69, 35.17, 28.17, 28.14.

*3-(4-Chloro-phenylamino)-propionic acid tert-butyl ester* (**3v**): The product was obtained as a white solid in a yield of 83% (0.53 g). MP: 33–36 °C. ^1^H NMR (600 MHz, CDCl_3_) δ 7.13 (d, *J* = 8.7 Hz, 2H), 6.57 (d, *J* = 8.7 Hz, 2H), 4.41 (s, 1H), 3.37 (t, *J* = 6.3 Hz, 2H), 2.52 (t, *J* = 6.3 Hz, 2H), 1.45 (s, 9H). ^13^C NMR (151 MHz, CDCl_3_) δ 171.60, 146.03, 129.13, 122.51, 114.40, 81.07, 39.98, 34.76, 28.11.

*3-(Methyl-phenyl-amino)-propionamide* (**3w**): The product was obtained as a white solid in a yield of 80% (0.43 g). MP: 88–90 °C. ^1^H NMR (600 MHz, DMSO-d6) δ 7.38 (s, 1H), 7.21 – 7.10 (m, 2H), 6.85 (s, 1H), 6.70 (d, *J* = 8.0 Hz, 2H), 6.65 – 6.54 (m, 1H), 3.56 – 3.49 (m, 2H), 2.84 (s, 3H), 2.29 – 2.09 (m, 2H). ^13^C NMR (151 MHz, DMSO-d6) δ 173.82, 149.12, 129.54, 116.60, 116.13, 112.78, 112.43, 48.84, 38.13, 32.50.

*3-(pyrrolidin-1-yl)propanamide* (**3x**) [37]: The product was obtained as a yellow liquid in a yield of 96% (0.48 g). ^1^H NMR (600 MHz, CDCl_3_) δ 8.21 (s, 1H), 5.88 (s, 1H), 2.79 (s, 2H), 2.62 (s, 4H), 2.45 (s, 2H), 1.82 (t, *J* = 3.5 Hz, 4H). ^13^C NMR (151 MHz, CDCl_3_) δ 175.51, 53.84, 53.43, 51.62, 34.13, 23.50, 22.75.

*3-(4-methylpiperidin-1-yl)propanamide* (**3y**): The product was obtained as a white solid in a yield of 95% (0.47 g). MP: 116–117 °C [38]. ^1^H NMR (600 MHz, CDCl_3_) δ 8.24 (s, 1H), 5.43 (s, 1H), 2.93 (d, *J*=11.7, 2H), 2.61 (td, *J* = 6.4, 3.8, 2H), 2.40 (t, *J* = 6.2, 2H), 2.00 (t, *J* = 11.0, 2H), 1.68–1.56 (m, 2H), 1.48–1.30 (m, 1H), 1.21 (ddd, *J* = 15.6, 12.5, 3.7, 2H), 0.88 (d, *J* = 6.5, 3H). ^13^C NMR (151 MHz, CDCl_3_) δ 174.94, 53.92, 53.27, 42.62, 37.18, 33.88, 31.92, 30.49, 21.63.

*Phenylamine* (**1a**) [39]: The product was obtained as a colorless liquid in a yield of 63% (0.12 g). ^1^H NMR (600 MHz, CDCl_3_) δ 7.11 (dd, *J* = 8.4, 7.4 Hz, 2H), 6.72 (t, *J* = 7.4 Hz, 1H), 6.58 (dd, *J* = 8.5, 1.0 Hz, 2H), 3.49 (s, 2H). ^13^C NMR (151 MHz, CDCl_3_) δ 146.69, 129.47, 118.61, 115.29.

*o-phenylenediamine* (**1b**): The product was obtained as a colorless solid in a yield of 82% (0.17 g). MP: 100–102 °C [40].^1^H NMR (600 MHz, CDCl_3_) δ 6.87 – 6.50 (m, 4H), 3.35 (s, 4H). ^13^C NMR (151 MHz, CDCl_3_) δ 134.71, 120.22, 116.70.

*1-Aminonaphthalene* (**1c**): The product was obtained as a white solid in a yield of 85% (0.2 g). MP: 45–48 °C [41]. ^1^H NMR (600 MHz, CDCl_3_) δ 7.79 (tt, *J* = 7.0, 4.2 Hz, 2H), 7.51 – 7.37 (m, 2H), 7.37 – 7.24 (m, 2H), 6.76 (dd, *J* = 7.1, 1.2 Hz, 1H), 4.12 (s, 2H). ^13^C NMR (151 MHz, CDCl_3_) δ 142.10, 134.43, 128.59, 126.38, 125.89, 124.90, 123.69, 120.83, 119.02, 109.72.

*4-Methoxy-phenylamine* (**1d**): The product was obtained as a brown solid in a yield of 72% (0.16g). MP: 56–59 °C [42]. ^1^H NMR (600 MHz, CDCl_3_) δ 6.86 – 6.68 (m, 2H), 6.68 – 6.57 (m, 2H), 3.85 – 3.61 (m, 3H), 3.39 (s, 2H). ^13^C NMR (151 MHz, CDCl_3_) δ 152.78, 139.92, 116.41, 114.79, 55.72.

*2-Chloro-phenylamine* (**1e**) [43]: The product was obtained as a colorless liquid in a yield of 40% (0.09 g). ^1^H NMR (600 MHz, CDCl_3_) δ 7.31 – 7.19 (m, 1H), 7.04 (dd, *J* = 10.2, 4.2 Hz, 1H), 6.68 (dd, *J* = 16.0, 6.6 Hz, 2H), 4.00 (s, 2H). ^13^C NMR (151 MHz, CDCl_3_) δ 143.12, 129.53, 127.82, 119.32, 119.12, 116.06.

*4-Bromo-phenylamine* (**1f**): The product was obtained as a grey solid in a yield of 70% (0.19 g). MP: 59–62 °C [44]. ^1^H NMR (600 MHz, CDCl_3_) δ 7.29 – 7.09 (m, 2H), 6.61 – 6.42 (m, 2H), 3.66 (d, *J* = 36.7 Hz, 2H). ^13^C NMR (151 MHz, CDCl_3_) δ 145.41, 131.95, 116.68, 110.09.

*Phenylamine* (**1g**): The product was obtained as a colorless liquid in a yield of 73% (0.12 g). ^1^H NMR (600 MHz, CDCl_3_) δ7.11 (dd, *J* = 8.4, 7.4 Hz, 2H), 6.72 (t, *J* = 7.4 Hz, 1H), 6.58 (dd, *J* = 8.5, 1.0 Hz, 2H), 3.49 (s, 2H). ^13^C NMR (151 MHz, CDCl_3_) δ 146.69, 129.47, 118.61, 115.29.

*4-Bromo-phenylamine* (**1h**): The product was obtained as a grey solid in a yield of 70% (0.17 g). MP: 59––62 °C. ^1^H NMR (600 MHz, CDCl_3_) δ 7.29 – 7.09 (m, 2H), 6.61 – 6.42 (m, 2H), 3.66 (d, *J* = 36.7 Hz, 2H). ^13^C NMR (151 MHz, CDCl_3_) δ 145.41, 131.95, 116.68, 110.09.

*o-phenylenediamine* (**1i**): The product was obtained as a colorless solid in a yield of 81% (0.19 g). MP: 100–102 °C. ^1^H NMR (600 MHz, CDCl_3_) δ 6.87 – 6.50 (m, 4H), 3.35 (s, 4H). ^13^C NMR (151 MHz, CDCl_3_) δ 134.71, 120.22, 116.70.

*3-Chloro-phenylamine* (**1j**) [45]: The product was obtained as a colorless liquid in a yield of 35% (0.09 g). ^1^H NMR (600 MHz, CDCl_3_) δ 7.01 (t, *J* = 8.0 Hz, 1H), 6.69 (dd, *J* = 7.9, 1.1 Hz, 1H), 6.59 (t, *J* = 2.1 Hz, 1H), 6.47 (dd, *J* = 8.1, 2.2 Hz, 1H), 3.65 (s, 2H). ^13^C NMR (151 MHz, CDCl_3_) δ 147.85, 134.82, 130.47, 118.42, 114.97, 113.36.

*p-Tolylamine* (**1k**): The product was obtained as a colorless solid in a yield of 72% (0.17 g). MP: 43–45 °C [46]. ^1^H NMR (600 MHz, CDCl_3_) δ 7.07 – 6.83 (m, 2H), 6.58 (d, *J* = 8.0 Hz, 2H), 3.46 (s, 2H), 2.23 (s, 3H). ^13^C NMR (151 MHz, CDCl_3_) δ 143.91, 129.82, 127.80, 115.32, 20.52.

*p-Tolylamine* (**1l**): The product was obtained as a colorless solid in a yield of 76% (0.18 g). MP: 43–45 °C. ^1^H NMR (600 MHz, CDCl_3_) δ 7.07 – 6.83 (m, 2H), 6.58 (d, *J* = 8.0 Hz, 2H), 3.46 (s, 2H), 2.23 (s, 3H). ^13^C NMR (151 MHz, CDCl_3_) δ 143.91, 129.82, 127.80, 115.32, 20.52.

*Phenylamine* (**1m**): The product was obtained as a colorless liquid in a yield of 72% (0.16 g). ^1^H NMR (600 MHz, CDCl_3_) δ7.11 (dd, *J* = 8.4, 7.4 Hz, 2H), 6.72 (t, *J* = 7.4 Hz, 1H), 6.58 (dd, *J* = 8.5, 1.0 Hz, 2H), 3.49 (s, 2H). ^13^C NMR (151 MHz, CDCl_3_) δ 146.69, 129.47, 118.61, 115.29.

*Chloro-phenylamine* (**1o**): The product was obtained as a white solid in a yield of 82% (0.21 g). MP: 65–67 °C [47]. ^1^H NMR (600 MHz, CDCl_3_) δ 7.09 (d, *J* = 8.6 Hz, 2H), 6.60 (d, *J* = 8.6 Hz, 2H), 3.64 (s, 2H). ^13^C NMR (151 MHz, CDCl_3_) δ 144.97, 129.14, 123.16, 116.25.

*2,3,4,5-tetrahydro-1H-1,5-benzodiazepin-2-one* (**6a, 6b** and **6c**): The products were obtained as a tan solid in yields of 74% (0.24 g), 68% (0.22 g) and 34% (0.17 g), respectively. MP: 138–140 °C [48,49]. ^1^H NMR (600 MHz, CDCl_3_) δ 8.36 (s, 1H), 6.96 (t, *J* = 7.5 Hz, 1H), 6.90 (d, *J* = 7.8 Hz, 1H), 6.82 (t, *J* = 7.5 Hz, 1H), 6.74 (d, *J* = 7.9 Hz, 1H), 3.70 – 3.63 (m, 2H), 2.80 – 2.69 (m, 2H).^13^C NMR (151 MHz, CDCl_3_) δ 174.06, 138.75, 126.36, 125.36, 122.19, 120.57, 119.89, 45.55, 36.19.

*4-Methyl-4,5-dihydro-1H-benzo[b][1,4]diazepin-2(3H)-one* (**6d**): The product was obtained as a white solid in a yield of 93% (0.30 g). MP: 182–184 °C [50,51]. ^1^H NMR (600 MHz, CDCl_3_) δ 8.60 (s, 1H), 7.01 – 6.97 (m, 1H), 6.95 (dd, *J* = 7.7, 1.0, 1H), 6.88 (t, *J* = 7.5, 1H), 6.78 (d, *J* = 7.8, 1H), 4.03 (dt, 1H), 3.55 (s, 1H), 2.64 (dd, *J* = 13.6, 4.0, 1H), 2.45 (dd, *J* = 13.7, 7.9, 1H), 1.33 (d, *J* = 6.3, 3H). ^13^C NMR (151 MHz, CDCl_3_) δ 173.07, 138.25, 128.00, 125.64, 122.19, 121.41, 121.00, 54.14, 41.44, 23.62.

*7,8-difluoro-2-methyl-1,2,3,5-tetrahydro-1,5-benzodiazepin-4-one* (**6e**): The product was obtained as a white solid in a yield of 90% (0.29 g). MP: 193.5–195 °C. ^1^H NMR (600 MHz, CDCl_3_) δ 8.46 (s, 1H), 6.82 (dd, *J* = 10.6, 7.9, 1H), 6.64 (dd, *J* = 11.0, 7.5, 1H), 4.05 (dd, *J* = 12.1, 6.3, 1H), 3.38 (s, 1H), 2.63 (dd, *J*=13.5, 4.5, 1H), 2.40 (dd, *J* = 13.5, 7.6, 1H), 1.72 (s, 1H), 1.33 (d, *J* = 6.3, 3H).^13^C NMR (151 MHz, CDCl_3_) δ = 172.99, 148.33, 148.24, 146.69, 146.60, 145.90, 145.84, 144.30, 144.21, 134.80, 124.80, 111.04, 110.91, 109.69, 109.56, 55.07, 40.76, 23.43.

*2,3-dihydro-1,5-benzothiazepine-4(5H)-one* (**6f**): The product was obtained as a brown solid in a yield of 34% (0.11 g). Mp: 215–216°C[52]. ^1^H NMR (600 MHz, CDCl_3_) δ 8.55 (s, 1H), 7.61 (d, *J* = 7.7, 1H), 7.37 (td, *J* = 7.7, 1.2, 1H), 7.17 (t, *J* = 7.6, 1H), 7.14 (d, *J* = 7.8, 1H), 3.46 (t, *J* = 6.9, 2H), 2.65 (t, *J* = 6.9, 2H).^13^C NMR (151 MHz, CDCl_3_) δ 173.71, 141.41, 135.51, 129.83, 126.97, 126.49, 123.30, 34.29, 33.54.

*3-Methyl-2,3-dihydrobenzo[b][1,4]thiazepin-4(5H)-one* (**6g**): The product was obtained as a white solid in a yield of 31% (0.10 g). Mp: 176–178 °C [53]. ^1^H NMR (600 MHz, CDCl_3_) δ 8.32 (s, 1H), 7.60 (d, *J* = 7.6, 1H), 7.36 (t, *J* = 6.9, 1H), 7.17 (t, *J* = 8.2, 1H), 7.13 (d, *J* = 7.8, 1H), 3.49 (dd, *J* = 11.3, 6.0, 1H), 3.05 – 2.98 (m, 1H), 2.79 (dt, *J* = 12.6, 6.3, 1H), 1.19 (d, *J* = 6.6, 3H).^13^C NMR (151 MHz, CDCl_3_) δ 175.74, 141.12, 135.15, 129.72, 127.72, 126.46, 123.47, 41.51, 36.33, 15.47.

## Figures and Tables

**Table 1:**
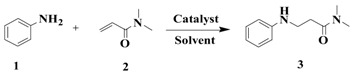
Optimization of conditions for the reaction of the Aza-Michael addition.

Entry	Cat (eq)	Solvent	Temperature (°C)	Time (h)	Yield (%) ^c^
1	-	-	120	4	trace
2	Imidazolium chloride (0.3)	-	120	4	69
3	Imidazole (0.3)	-	120	6	10
4	HCl (0.3)	-	120	4	60
5	Imidazolium chloride (0.3)	-	90	6	50
6	Imidazolium chloride (0.3)	-	120	4	75
7	Imidazolium chloride (0.3)	-	130	4	65
8	Imidazolium chloride (0.3)	-	140	4	40
9	Imidazolium chloride (0.1)	-	120	4	32
10	Imidazolium chloride (0.2)	-	120	4	61
11	Imidazolium chloride (0.5)	-	120	4	73
12 ^b^	Imidazolium chloride (0.3)	H_2_O	100	6	18
13 ^b^	Imidazolium chloride (0.3)	CH_3_CN	70	6	NO
14 ^b^	Imidazolium chloride (0.3)	Toluene	120	4	63
15 ^b^	Imidazolium chloride (0.3)	Xylene	120	4	66

^a^ Reaction conditions: Unless otherwise noted, all reactions were carried out with **1** (10.8 mmol, 1 equiv), **2** (14.0 mmol, 1.3 equiv), Imidazolium chloride (0.1–0.5 equiv); ^b^ All solvents are 2mL; ^c^ Yields of products **3**. Abbreviations: NO, no reaction.

**Table 2:**
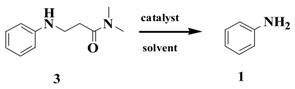
Optimization of reaction conditions for the deprotection of amino group ^a^.

Entry	Cat (eq)	Solvent	Temperature (°C)	Time (h)	Yield (%) ^b^
1	-	-	150	6	Trace
2	Imidazole (0.7)	-	150	6	Trace
3	HCl (0.7)	-	150	6	8
4	Imidazolium chloride (0.7)	-	120	6	Trace
5	Imidazolium chloride (0.7)	-	150	6	61
6	Imidazolium chloride (0.7)	-	160	6	63
7	Imidazolium chloride (0.7)	-	180	6	65
8	Imidazolium chloride (0.1)	-	150	6	23
9	Imidazolium chloride (0.5)	-	150	6	35
10	Imidazolium chloride (1.0)	-	150	6	65
11 ^c^	Imidazolium chloride (0.7)	H_2_O	100	6	NO
12 ^c^	Imidazolium chloride (0.7)	benzene	90	6	NO
13 ^c^	Imidazolium chloride (0.7)	xylene	150	6	57

^a^ General conditions: **3** (5.2 mmol, 1 equiv), imidazolium chloride (0.1–1 equiv) under solvent-free conditions for 6 h; ^b^ Isolated yields are given; ^c^ All solvents are 2 mL. Abbreviations: NO, no reaction.

**Table 3:**
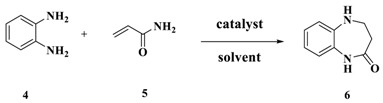
Optimization of synthesis of medium ring heterocycles ^a^.

Entry	Cat(eq)	Solvent	Temp(°C)	Time(h)	Yield(%) ^b^
1	Imidazolium chloride(0.3)	xylene	120	5	45
2	-	xylene	120	5	NO
3	HCl(0.3eq)	xylene	120	5	34
4	Imidazolium(0.3)	xylene	120	5	trace
5	Imidazolium chloride(0.3)	xylene	80	5	13
6	Imidazolium chloride(0.3)	xylene	100	5	25
7	Imidazolium chloride(0.3)	xylene	110	5	31
8	Imidazolium chloride(0.3)	xylene	140	5	75
9	Imidazolium chloride(0.3)	xylene	150	5	76
10	Imidazolium chloride(0.3)	CH_3_CN	70	9	15
11	Imidazolium chloride(0.3)	H_2_O	100	9	10
12	Imidazolium chloride(0.3)	dioxane	100	9	27
13	Imidazolium chloride(0.3)	toluene	110	9	10
14	Imidazolium chloride(0.3)	ethylene glycol diethyl ether	120	5	39
15	Imidazolium chloride(0.3)	2-methoxyethanol	125	5	43
16	Imidazolium chloride(0.3)	2-ethoxyethanol	140	5	trace
17	Imidazolium chloride(0.1)	xylene	140	5	19
18	Imidazolium chloride(0.5)	xylene	140	5	49

^a^ General conditions: **4** (4.6 mmol, 1 equiv), **5** (7.9 mmol, 1.7 equiv), imidazolium chloride (0.1–0.5 equiv) under solvent-free conditions for 6 h; ^b^ Isolated yields are given. All solvents are 2 mL. Abbreviations: NO, no reaction.

**Table 4:** Substrate scope of substituted amines and acrylamide derivatives ^a^.

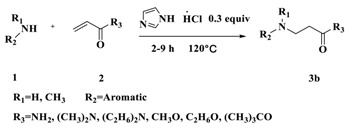
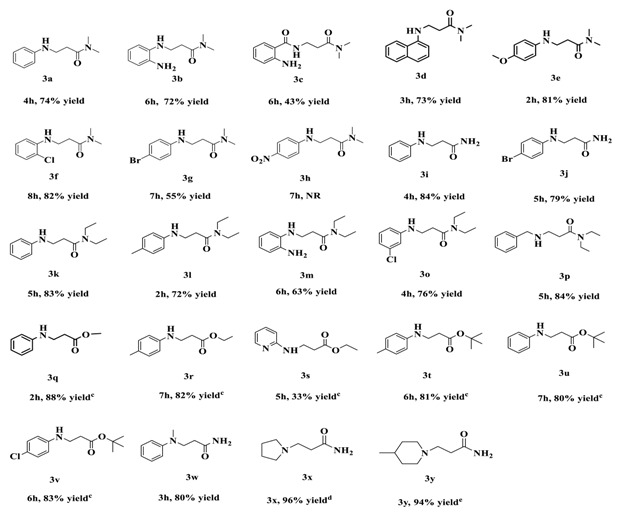

^a^ Reaction conditions: Unless otherwise noted, **1** (5 mmol, 1 equiv), **2** (6.5 mmol, 1.3 equiv), imidazolium chloride (0.3 equiv), solvent-free conditions, 2–9 h, at 120 °C; ^b^ Yields after column purification; ^c^ This reaction was performed at 100 °C; ^d^ This reaction was performed for 0.5 h at rt. ^e^ This reaction was performed for 10 min at 70 °C. Abbreviations: NR, no reaction.

**Table 5:** Substrate scope of the deprotection of aromatic amino groups ^a^.

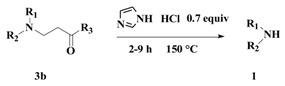				
Entry	Substance	Time (h)	Product	Yield ^b^ (%)
1	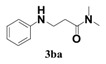	6	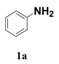	63
2	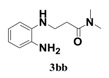	4	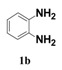	82
3	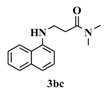	9	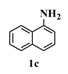	85
4	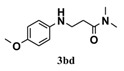	5	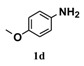	72
5	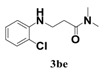	12	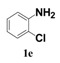	40
6	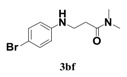	11	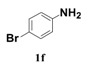	70
7	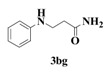	10	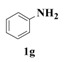	73
8	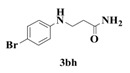	6	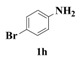	70
9	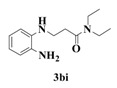	4	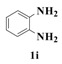	81
10	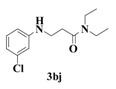	10	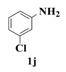	35
11	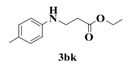	9	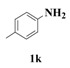	72
12	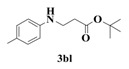	6	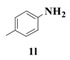	76
13	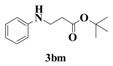	6	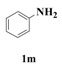	72
14	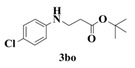	6	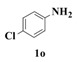	82

^a^ Reaction conditions: Unless otherwise noted, All reactions were carried out with **3b** (1.0 mmol, 1 equiv) and imidazolium chloride (0.7 mmol, 0.7 equiv), under solvent-free conditions for 4–11 h at 150 °C; ^b^ Yields after column purification. Abbreviations: NR, no reaction.

**Table 6:** Substrate scope of substituted 1,3,4,5-tetrahydro-2H-1,5-benzodiazepine-2-ones and substituted 2,3-dihydrobenzo[b][1,4]thiazepin-4(5H)-ones ^a^.

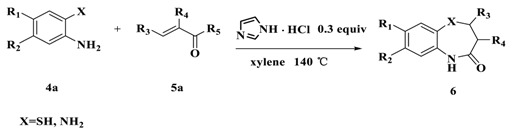					
Entry	Substance		Time (h)	Product	Yield ^b^ (%)
1	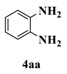	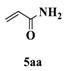	5	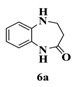	74
2	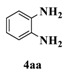	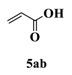	5	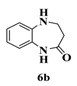	68
3 ^c^	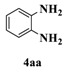	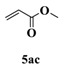	13	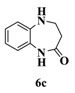	34
4	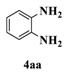	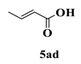	0.5	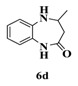	93
5	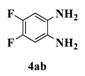	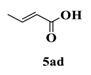	4	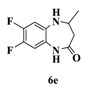	90
6	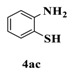	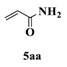	2	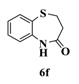	34
7	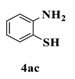	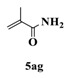	2	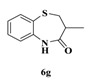	31

^a^ Reaction conditions: Unless otherwise noted, **4a** (3.0 mmol, 1 eq) and **5a** (5.1 mmol, 1.7 eq), imidazolium chloride (0.3 equiv), at 140 °C; ^b^ Yields after column purification; ^c^ This reaction was performed at 90 °C for 5 h and then at 140 °C for 8 h. Abbreviations: NR, no reaction.

## Supplementary Materials

The following are available online at https://www.mdpi.com/1420-3049/24/23/4224/s1. Figures S1–S74: C-NMR and H-NMR spectra of the products.
